# Is laterality in breast Cancer still worth studying? Local experience in Bahrain

**DOI:** 10.1186/s12885-022-10063-y

**Published:** 2022-09-10

**Authors:** Suhair Al Saad, Hamdi Al Shenawi, Amer Almarabheh, Noor Al Shenawi, Abdulla Ismaeel Mohamed, Rami Yaghan

**Affiliations:** 1 Department of Surgery, College of Medicine and Medical Sciences, Arabian Gulf University, Manama, Bahrain; 2 Department of Family and Community Medicine, College of Medicine and Medical Sciences, Arabian Gulf University, Manama, Bahrain; 3 Undergraduate medical student, College of Medicine and Medical Sciences, Arabian Gulf University, Manama, Bahrain

**Keywords:** Breast cancer 1, Laterality 2, Bahrain 3, Tumor stage 4, Family history 5, Size of tumor 6

## Abstract

**Background:**

Laterality in breast cancer means an increased frequency of left-sided breast cancers compared to right-sided breast cancers ranging between 1.05 and 1.26. It was first described in 1935 by Fellenberg, Sweden. The explanation of this phenomenon is not clear, but the association with other factors was found. This study aimed to explore the laterality of breast cancer in Bahrain as a model for Arabian countries. The association of laterality with the clinicopathological characteristics of the tumor was also analyzed to explore any applied clinical value.

**Methods:**

This is a cross-sectional, retrospective review of a particular ethnic population to study laterality of breast cancer versus a number of clinicopathological factors, as well as prognosis. The study analyzed 228 breast cancer patients treated in Arabian Gulf University facilities in Bahrain between 1999 and 2020. Three bilateral breast cancer and two malignant phyllodes patients were excluded.

The following variables were analyzed: laterality ratio (Lt/Rt) and the association between laterality and clinicopathological characteristics (age at diagnosis, family history of malignancy, size of the tumor, tumor grade, histological type, hormonal receptors and HER2, axillary lymph node status, tumor stage, five-year survival rate, nulliparity, and multifocality).

**Results:**

The laterality ratio (Lt/Rt) was 1.06 and was 0.97 for patients below 50 years of age, and 1.19 for patients 50 years of age and above.

Analysis of our data showed a statistically significant association between laterality and tumor stage (*p*. value =0.025) at presentation, and laterality and family history of malignancy (*p*. value =0.052).

Right-sided breast cancer was associated with a higher positive family history of malignancy and an increased ratio of locally advanced and metastatic disease, and a reduced 5-year survival in relation to size and stage. Left-sided breast cancer was associated with higher early tumor stage.

**Conclusion:**

This is the first study exploring the issue of breast cancer laterality in a defined Arabian population. The laterality ratio in this study was 1.06, which is consistent with the globally published range (1.05 to 1.26) and is increasing with increasing age.

The association between breast cancer laterality, and the hormonal and HER2 is still not widely addressed in the available literature, although other clinicopathological characteristics were extensively analyzed.

## Background

Laterality is a recognized fact in breast cancer patients [1**,** 2]. Left-sided breast cancer incidence is higher than right-sided breast cancer in a ratio varying from 1.05 to 1.26 [1]. Fellenberg was one of the first to recognize the left-sided predilection of breast cancer while analyzing the 1933–1935 Swiss Cancer Census [2]. Although many theories were proposed to explain this side disparity, no clear explanation has been achieved [1**–**3]. One study suggested that the tissue mass in paired organs, like the breasts, is asymmetrical and more tissue is located in the left breast, contributing to the mild left-sided predilection of breast cancer [2**,** 4]. Few studies reported some interesting observations related to the side of breast cancer and the side of the dominant hand. In this regard, one study reported that the onset of breast cancer among left-handed patients presents 2 years earlier compared to right-handed patients. They suggested that it was linked to higher fetal estrogen exposure in left-handed patients and advised that screening mammography should start at an earlier age in left-handed patients [5**,** 6]. Ramadhani reported higher mortality from breast cancer and colorectal cancer among left-handed patients, although this was not a universal finding in other studies [7**,** 8].

### The aim of the study

This study aimed to explore laterality of breast cancer in Bahrain as a model for Arabian countries. These countries share peculiar characteristics (younger age at diagnosis, higher percentage of positive family history of malignancy, and higher ratio of premenopausal breast cancer). The association between laterality and the pertinent tumor clinicopathological characteristics, if any, was also analyzed to explore any applied clinical value. The following clinicopathological characteristics were considered in this regard: age at diagnosis, nulliparity, breastfeeding history, family history, size of the primary tumor, tumor grade, histological type of breast cancer, hormonal receptors, HER2, multifocality, lymph node status, presence of metastasis at diagnosis, tumor stage, and the five-year survival rate.

## Methods

### Design and setting of the study

This is a cross-sectional, retrospective review of a particular ethnic population to study laterality of breast cancer versus a number of clinicopathological factors, as well as prognosis. The study analyzed breast cancer female patients treated by the first, second, and sixth authors between August 1999 and October 2020. All patients were treated in governmental and private hospitals affiliated to the Arabian Gulf University (AGU) (Salmaniya Medical Complex, Al Kindi Hospital and Al Salam Specialist Hospital).

Patients whom our group operated upon during this period were added consecutively to the study group. All non-Bahraini female patients were excluded. Only 228 Bahraini patients with malignant breast lesions were included. 3 patients with bilateral synchronous invasive ductal carcinoma (IDC), and 2 patients with malignant phyllodes tumors were excluded from the study. The remaining 223 patients with unilateral breast carcinomas were the subject of this study. The following variables were analyzed: laterality ratio, the association between tumor laterality and: age category at diagnosis (below 50 years and above or equal to 50 years), nulliparity (yes or no), history of breastfeeding (yes or no), presence of positive family history of cancer (breast, colon, ovarian) - (yes or no), size of the primary tumor at presentation (below or equal to 5 cm and above 5 cm), tumor grade (G1,G2 and G3), histopathology of the primary tumor [IDC no special type (NST) and special type (ST), Ductal carcinoma in situ (DCIS), Invasive lobular carcinoma (ILC), Mixed Invasive ductal & lobular carcinoma], Estrogen (ER) and Progesterone (PR) hormonal receptors status (positive or negative), HER2 (positive or negative), multifocality (yes or no), axillary lymph node status (positive or negative), tumor stage at diagnosis [early (T1, T2, N0-N1, M0), locally advanced (T3, T4, N1-N3, M0), and metastatic (M0 or M1)], and the five-year survival rate.

### Statistical analysis

The statistical analysis was conducted using the statistical package for social science (SPSS) software version 28. Frequencies and percentages were computed for the categorical variables. A pie chart was used to present a categorical variable. The number and ratio of left- to right-sided tumors were calculated according to different clinicopathological factors. Chi-Square test was used to determine whether there is a significant association between two categorical variables. Factors influencing survival were estimated by Kaplan–Meier survival analysis. The log-rank test was used to compare the distribution of survival between groups. In all analyses, a two-sided *p*. value less than 0.05 was considered statistically significant.

## Results

**Fig. 1:**
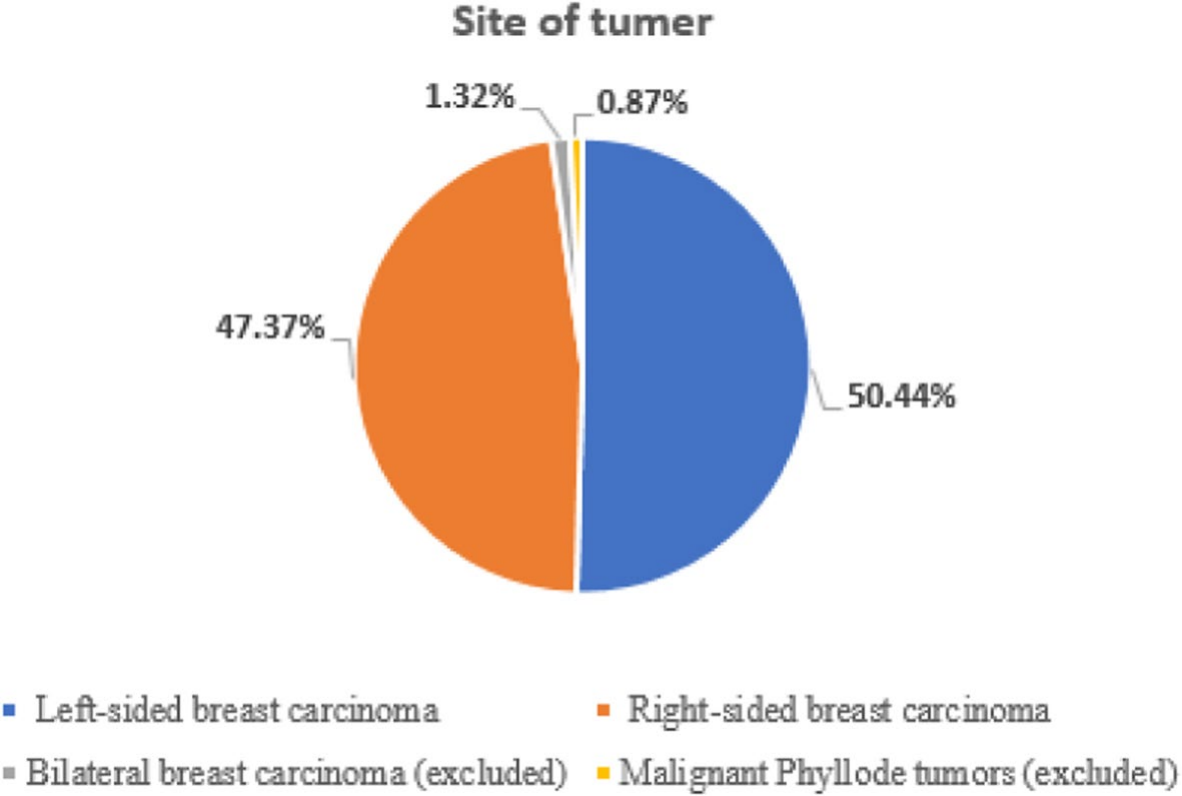
Site of tumor

Only 223 patients with unilateral breast carcinoma were included in our study. The number of left-sided breast cancer patients was 115 (50.44%). The number of right-sided breast cancer patients was 108 (47.37%). (Fig. 1)

### Laterality of breast cancer

The laterality ratio (Lt/Rt) in general was 1.06. We measured the laterality for patients below 50 years of age and was found to be 0.97, but for patients 50 years of age and above the laterality was higher or equal to 1.19. (Table 1).

**Table 1:** Laterality ratio according to age at diagnosis

	Right	Left	Laterality Ratio
All ages	108	115	1.06
< 50 years	60	58	0.97
≥50 years	48	57	1.19

Association between breast cancer laterality and Patient demographic data (Table 2).

**Table 2:** Patient demographic data in relation to breast cancer laterality (*n* = 223)

	Right-sided Breast n (%)	Left-sided Breast n (%)	*P*. value
Age at diagnosis			
< 50	60 (55.6)	58 (50.4)	0.444
≥ 50	48 (44.4)	57 (49.6)	
Nulliparous			
Yes	27 (25.0)	20 (17.4)	0.164
No	81 (75.0)	95 (82.6)	
History of breastfeeding			
Yes	74 (68.5)	86 (75.4)	0.251
No	34 (31.5)	28 (24.6)	
*Family history of malignancy*			
Yes	43 (40.2)	32 (27.8)	*0.052*
No	64 (59.8)	83 (72.2)	
Size of tumor			
≤ 5 cm	88 (81.5)	102 (88.7)	*0.129*
> 5 cm	20 (18.5)	13 (11.3)	
Summarized histopathology			
Invasive Ductal Carcinoma (NST)	91 (84.3)	84 (73.0)	0.212
Invasive Ductal Carcinoma (ST)	7 (6.5)	11 (9.6)	
Ductal carcinoma in situ (DCIS)	3 (2.8)	9 (7.8)	
Invasive Lobular Carcinoma (NST)	6 (5.6)	7 (6.1)	
Mixed Invasive Ductal & Lobular	1 (0.9)	4 (3.5)	
ER/PR Hormonal Status			
Positive	65 (61.9)	68 (60.7)	0.866
Negative	35 (33.3)	40 (35.7)	
Not available	5 (4.8)	4 (3.6)	
HER2			
Positive	33 (31.4)	30 (27.3)	0.494
Negative	61 (58.1)	72 (65.5)	
Not available	11 (10.5)	8 (7.3)	
Multifocal			
Yes	23 (21.3)	31 (27.4)	0.442
No	81 (75.0)	76 (67.3)	
Not available	4 (3.7)	6 (5.3)	
Final stage: lymph node			
Negative	47 (43.5)	57 (49.6)	0.366
Positive	61 (56.5)	58 (50.4)	
Final stage: metastasis			
Mo	93 (86.1)	104 (90.4)	0.315
M1	15 (13.9)	11 (9.6)	
*Tumor Stage*			
Early	70 (64.8)	93 (80.9)	*0.025*
Locally advanced	24 (22.2)	13 (11.3)	
Metastatic (M1)	14 (13.0)	9 (7.8)	
Five-year survival			
Yes	74 (79.6)	84 (82.4)	0.408
No	16 (17.2)	12 (11.8)	
Lost follow up	3 (3.2)	6 (5.9)	
Site of recurrence			
Local	7 (6.9)	5 (4.5)	0.199
Distant Metastasis	10 (9.9)	20 (18.0)	
No	84 (83.2)	86 (77.5)	
History of malignancy			
Yes	2 (1.9)	3 (2.6)	0.703
No	105 (98.1)	111 (97.4)	
Malignancy type in past			
Breast	0 (0)	1 (33.3)	0.172
Ovaries	1 (50.0)	0 (0)	
Uterine	0 (0)	2 (66.7)	
Others	1 (50.0)	0 (0)	
Grade of the main tumor			
Grade 1	10 (9.8)	10 (9.8)	0.608
Grade 2	50 (49.0)	47 (46.1)	
Grade 3	37 (36.3)	40 (39.2)	
Not available	5 (4.9)	5 (4.9)	
Vascular and lymphatic invasion			
Negative	50 (48.1)	46 (43.8)	0.775
Positive	52 (50.0)	56 (53.3)	
Not available	2 (1.9)	3 (2.9)	

The mean age for all patients was 49.59 years, SD (11.29). The mean age for right-sided cancer was 49.42 years, SD (11.66). The mean age for left-sided cancer was 49.76 years, SD (10.97). We observed that the incidence in the right-sided breast cancer patients with age less than 50 years was slightly higher (*n* = 60, 55.6%) than the age 50 years & above (*n* = 48, 44.4%), while, in the left-sided breast cancer patients, both age categories were almost similar.

Nulliparity (*n* = 47) was more common among patients with right-sided breast cancer (25% in the right-side compared to 17.4% in the left-side).

The number of patients who practiced breastfeeding was high (*n* = 160, 71.75%). History of breast feeding was slightly higher in left-sided breast cancer (*n* = 86, 75.4%) compared to the right-sided (*n* = 74, 68.5%).

Positive family history of malignancy (breast cancer, colon cancer, and ovarian cancer) was encountered in 75 patients (33.63%). It was higher in right-sided breast cancer patients (*n* = 43, 40.2%) compared to left-sided (*n* = 32, 27.8%).

Tumors less or equal to 5 cm in size occurring in the right-sided breast (*n* = 88, 81.5%) were lower than in the left-sided (*n* = 102, 88.7%). Tumors above 5 cm in size occurring in the right-sided breast cancer patients (*n* = 20,18.5%) were more common compared to the left-sided (*n* = 13, 11.3%).

IDC (NST) (*n* = 175) was the commonest histological subtype. IDC (NST) was higher in right-sided breast cancer patients (*n* = 91, 84.3%) than the left-sided (*n* = 84, 73%). IDC (ST) was (*n* = 18). IDC (ST) was lower in the right-sided breast cancer patients compared to the left-sided (*n* = 7, 6.5%) vs (*n* = 11, 9.6%). DCIS was present in 12 patients. DCIS was lower in the right-sided breast cancer patients compared to the left-sided (*n* = 3, 2.8%) vs (*n* = 9, 7.8%). ILC was found in 13 patients. ILC was almost similar between both sides, right-sided breast cancer patients were (*n* = 6, 5.6%) and left-sided were (*n* = 7, 6.3%). Mixed Invasive Ductal & Lobular was low and only (*n* = 5). It was lower in the right-sided breast cancer patients (*n* = 1, 0.9%) than the left-sided (*n* = 4, 3.5%).

HER2 was overexpressed in (*n* = 63, 28.25%) and negative in (*n* = 133, 59.64%). HER2 overexpression was slightly higher in the right-sided breast cancer patients (*n* = 33, 31.4%) than the left-sided (*n* = 30, 27.3%). The negative HER2 was higher in the left-sided breast cancer patients (*n* = 72, 65.5%) than the right-sided (*n* = 61, 58.1%).

Multifocality was present (*n* = 54, 24.22%) only, and the majority were not multifocal (*n* = 157, 70.4%). It was lower in the right-sided breast cancer patients (*n* = 23, 21.3%) than the left-sided (*n* = 31, 27.4%).

Positive lymph node status was (*n* = 119, 53.36%). It was higher in the right-sided breast cancer patients (*n* = 61, 56.5%) than the left-sided (*n* = 58, 50.4%).

Positive distant metastasis status (M1) was only (*n* = 26, 11.6%). It was higher in the right-sided breast cancer patients (*n* = 15, 13.9%) than the left-sided (*n* = 11, 9.6%).

Laterality in relation to tumor stage showed a significant statistical association (*p*. value = 0.025). The early breast cancer was (*n* = 163, 73.09%). The early breast cancer stage was lower in the right-sided breast cancer patients (*n* = 70, 64.8%) than the left-sided (*n* = 93, 80.9%). Locally advanced breast cancer was (*n* = 37, 16.59%). The locally advanced stage was higher in the right-sided breast cancer patients (*n* = 24, 22.2%) than the left-sided (*n* = 13, 11.3%). Metastatic breast cancer patients were (*n* = 23, 10.31%). The metastatic stage was higher in the right-sided breast cancer patients (*n* = 14, 13%) than the left-sided (*n* = 9, 7.8%).

The five-year survival rate in all stages of breast cancer was (70.85%). The five-year survival rate was lower in the right-sided breast cancer patients (*n* = 74, 79.6%) than the left-sided (*n* = 84, 82.4%).

Local recurrence was higher in the right-sided breast cancer patients (n = 7, 6.9%) than the left-sided (*n* = 5, 4.5%). Distant metastasis was lower in the right-sided breast cancer patients (*n* = 10,9.9%) than the left-sided (*n* = 20, 18.0%).

### Impact of laterality on survival

The average follow up was 138 months, and median follow up was 143.5 months.

**Fig. 2:**
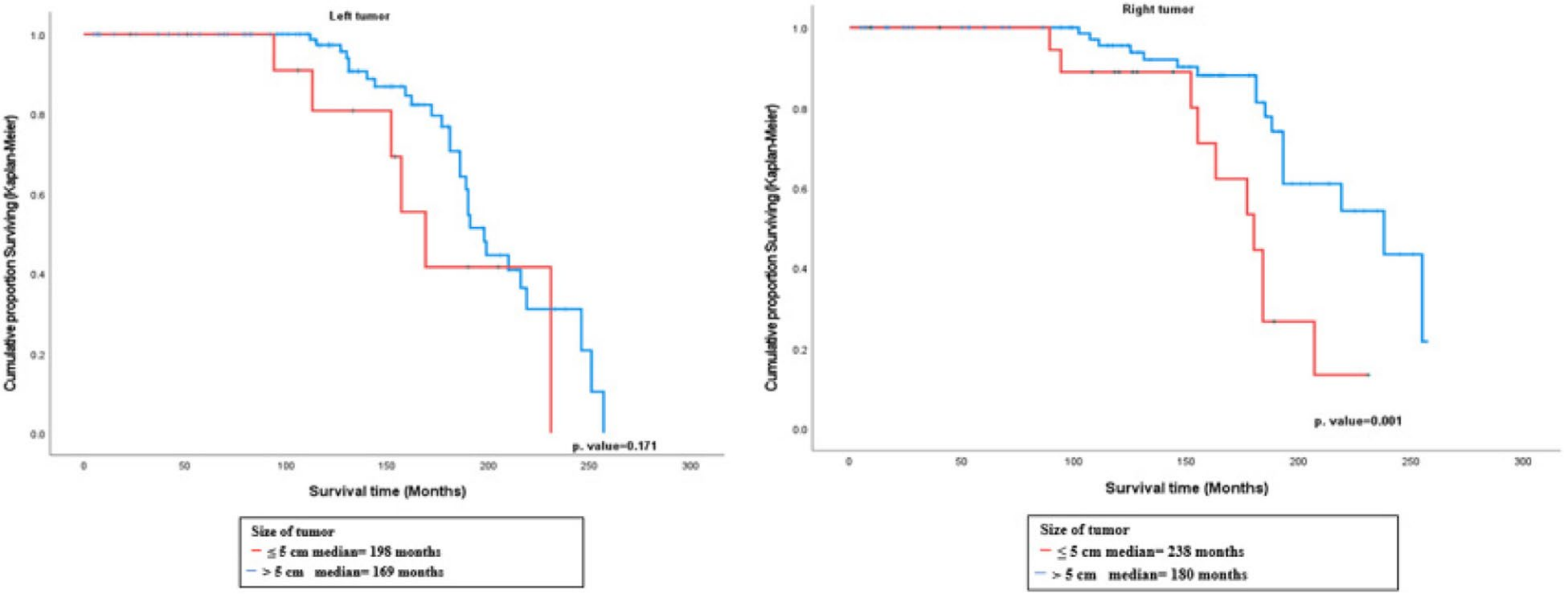
Plot of survival functions for breast cancer patients according to tumor size

The results of survival analysis using Kaplan–Meier showed that the median survival time for the right-sided breast cancer patients using interpolation method for the patients with tumor size ≤5 cm was around 238 months, whereas for tumor size > 5 cm was around 180 months. The Log-rank test showed a statistically significant difference in survival according to tumor size in the right-sided breast cancer patients (χ2 = 10.638, *p* < 0.001), while it was not statistically significant in the left-sided (≤ 5 cm, > 5 cm) (χ2 = 1.870, *p* = 0.171). (Fig. 2).

**Fig. 3:**
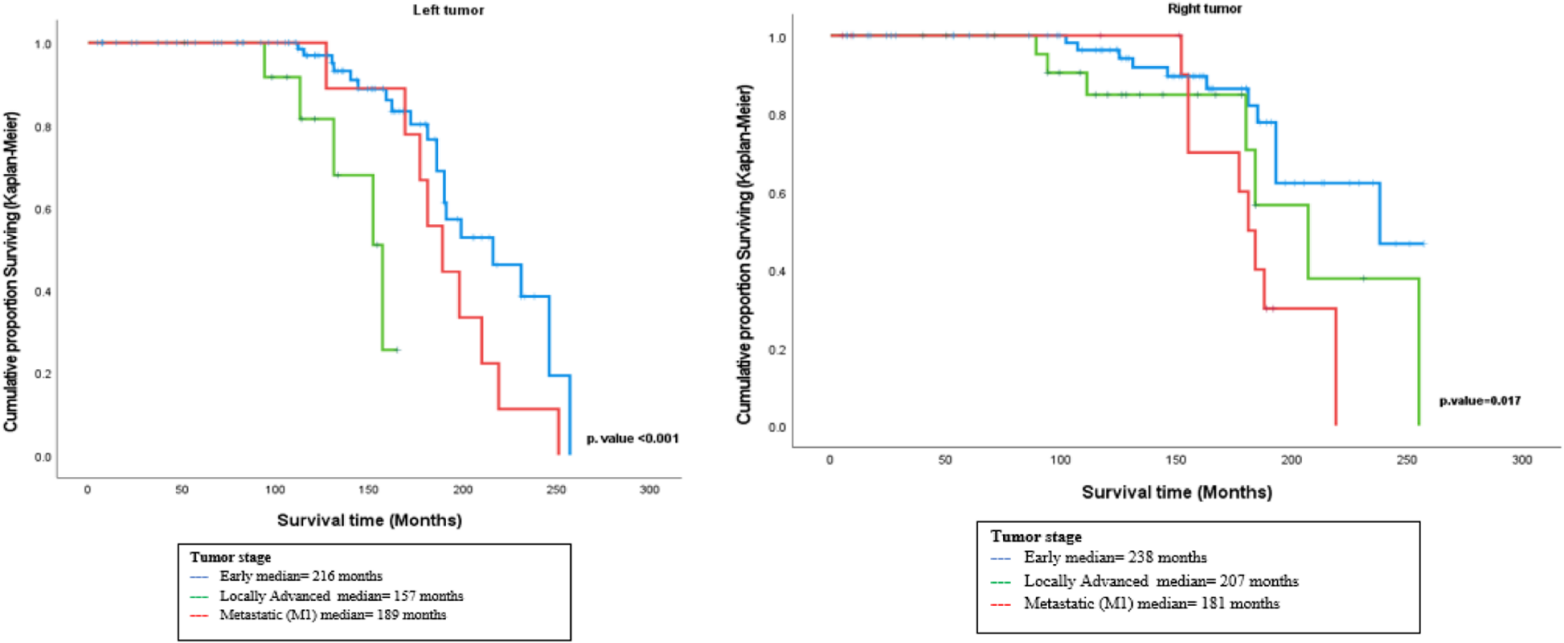
Plot of survival functions for breast cancer patients according to tumor stage

The results of survival analysis showed that the median survival time for the right-sided breast cancer patients who were early stage was around 238 months, and the median survival time for the patients who were locally advanced stage was around 207 months, whereas the median for the patients who had positive metastatic disease was around 181 months, and the results of the log-rank test indicated that there was a statistically significant difference in survival between patients according to tumor stage in the right-sided breast cancer patients (χ2 = 8.110, *p* = 0.017). (Fig. 3).

Also, the results related to tumor stage in the left-sided breast cancer patients indicated that the median survival time for the patients who were early stage was around 216 months, and the median survival time for the patients who were locally advanced stage was around 157 months, whereas the median for the patients who had positive metastatic was around 189 months, and the results of the log-rank test indicated that there was a statistically significant difference in survival between patients according to tumor stage in the left-sided breast cancer patients (χ2 = 16.950, *p* < 0.001). (Fig. 3).

**Fig. 4:**
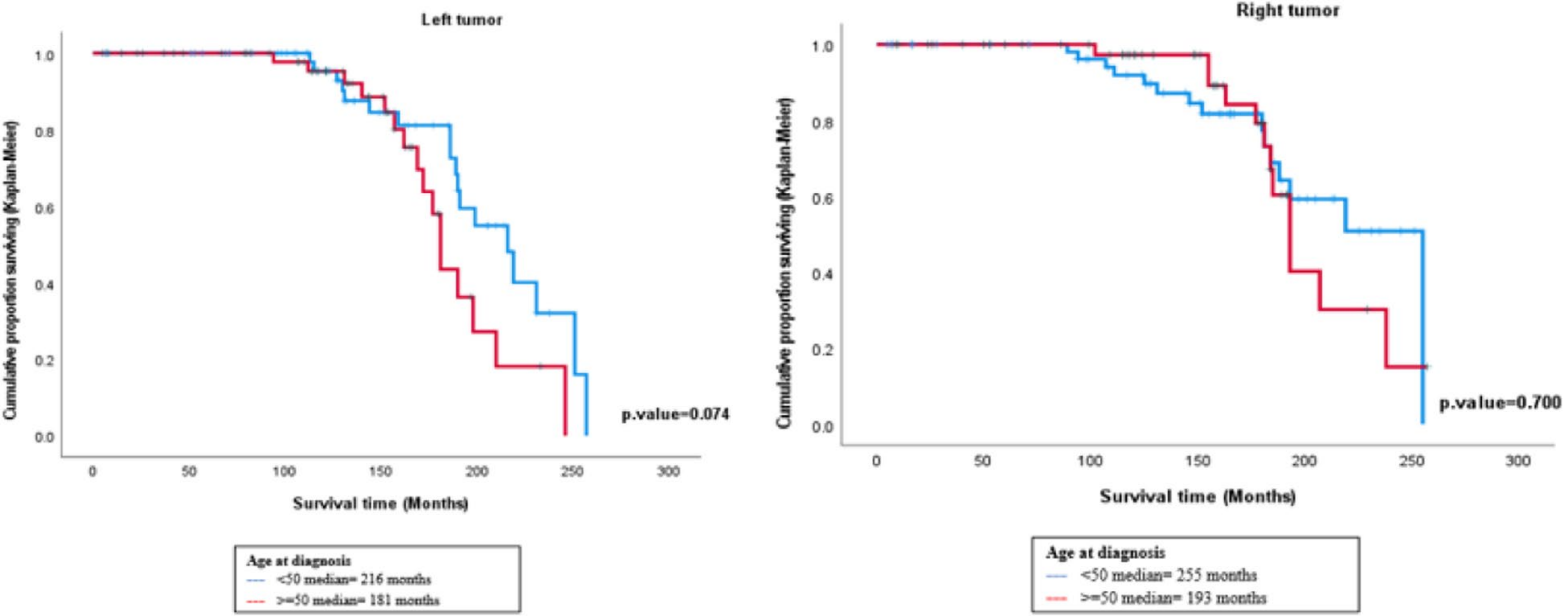
Plot of survival functions for breast cancer patients according to age at diagnosis

The results of survival analysis using Kaplan–Meier showed that the median survival time for the right-sided breast cancer patients using interpolation method for age < 50 years was around 255 months, whereas for age ≥ 50 years was around 193 months. The Log-rank test showed no statistically significant difference in survival between patients according to age in the right and the left-sided, respectively (χ2 = 0.149, *p* = 0.70) (χ2 = 3.191, *p* = 0.074). (Fig. 4).

**Fig. 5:**
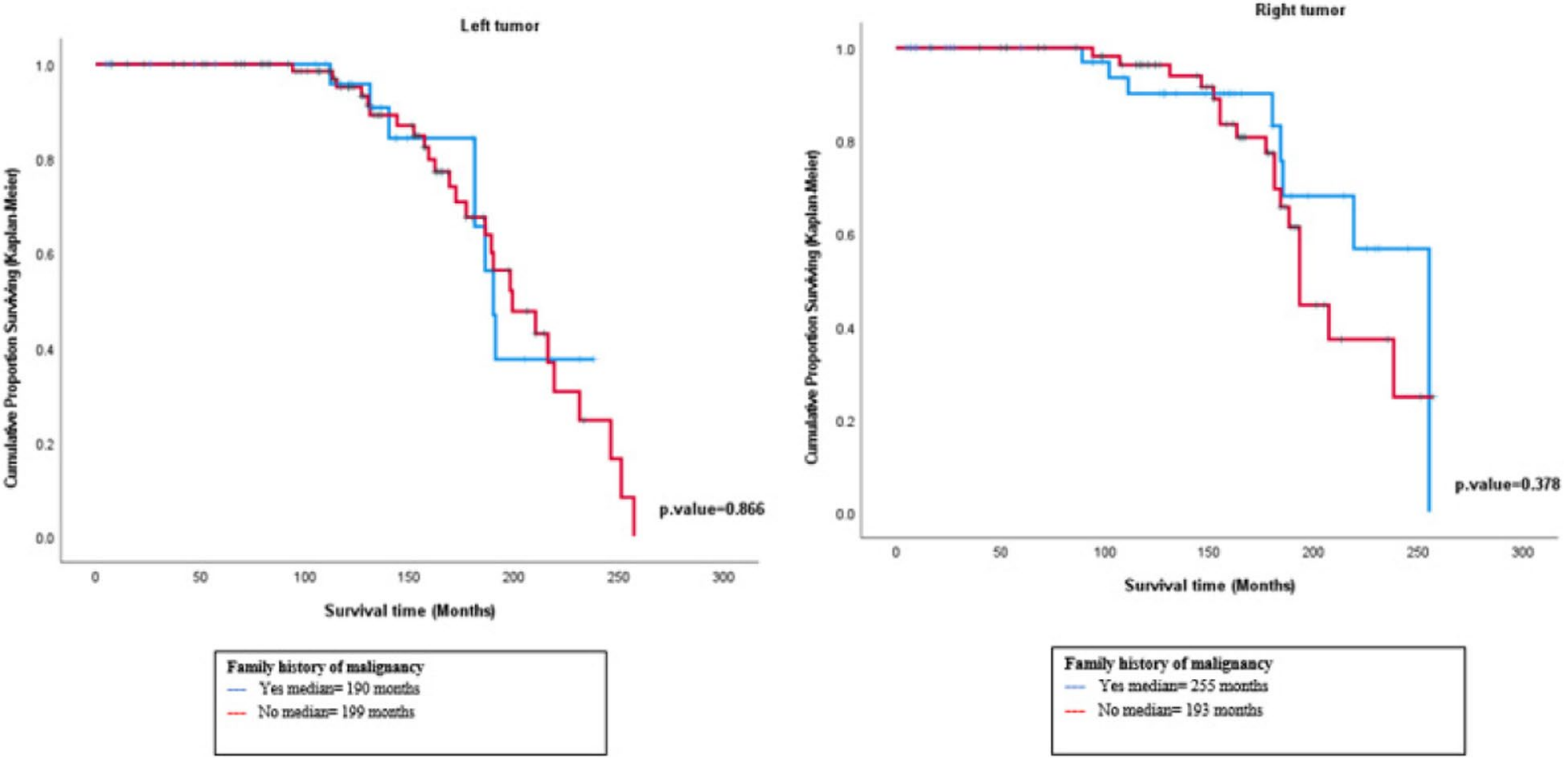
Plot of survival functions for breast cancer patients according to family history of malignancy

The results of survival analysis using Kaplan–Meier showed that the median survival time for the right-sided breast cancer patients using interpolation method for the patients with positive family history of malignancy was around 255 months, whereas the median for the patients with negative family history was around 193 months. The Log-rank test showed no statistically significant difference in survival between patients according to family history in the right and the left-sided, respectively (χ2 = 0.776, *p* = 0.378) (χ2 = 0.028, *p* = 0.866). (Fig. 5)

## Discussion

There is paucity of data in regard to laterality of breast cancer from the Middle East and Arabian Gulf countries. This study explored the issue of laterality in Bahraini female patients as a model for these countries, as they share common demographic characteristics. Such characteristics are well- demonstrated in our results and in previously published articles from Bahrain [9**,** 10]. These include younger age at diagnosis, a high percentage of positive family history of breast cancer, and a high ratio of premenopausal breast cancer [9**,** 10].

The left-sided predilection of breast cancer was recognized 70 years ago, [2] and confirmed by a substantial number of subsequent reports thereafter; the clinicopathological significance of this is still a matter of speculation. Table 3 illustrates the wide spectrum of similarities and differences in the clinicopathological characteristics of breast cancer in representative studies in relation to the issue of laterality.

**Table 3:** Laterality in breast cancer

References/Variables	Laterality	Right-sided breast cancer	Left-sided breast cancer
*Our study: Al Saad* et al*, 2022.*	-Laterality ratio is 1.06 (consistent with international reported range). -Laterality ratio was increasing with increasing age (1.187)	-Higher Family History (40.2%) -Locally advanced and distant metastatic tumor stages −5-year survival was statistically significant with size of tumor −5- year survival was statistically significant with stage of tumor -Age < 50 years −25% Nulliparity -Tumor size > 5 cm -Histopathology IDC mostly NST -Positive HER2 -Positive lymph node status -Higher local recurrence − 5- year survival was not statistically significant with age of patient − 5- year survival was not statistically significant with family history	-Lower Family history (27.8%) -Early tumor stage − 5-year survival was statistically significant with stage of tumor −17% Nulliparity -Higher breastfeeding history -Tumor size = or < 5 cm -Histopathology IDC ST and DCIS -Negative HER2 -Frequent multifocality -Higher systemic recurrence − 5-year survival was not statistically significant with size of tumor − 5- year survival was not statistically significant with age of patient − 5- year survival was not statistically significant with family history
Badru F et al., 201 1[1].	Positive laterality	Not Applicable	Right-handed patients find small lesions in the left-side breast earlier.
Senie RT et al., 198 0[2].	Positive laterality ratio of 1.26.	Not Applicable	-Significant association with clinicopathologic features: menarche after age 13, age at diagnosis, parity especially among those between ages 40–54, and all histologic types except medullary tumors. -Left-side breast has a 5% larger size
Weiss HA et al., 199 6[3].	-Positive laterality − 5% excess left-sided breast cancer, it increases with age.	Not Applicable	Left-side breast has slightly larger size
Roychoudhuri R et al., 200 6[4].	Breast laterality ratio is 1.07	Not Applicable	- Higher tissue mass in left-side breast which leads to increased cancer incidence
Cheng SA et al., 201 8[5].	Laterality ratio in age < 40 years is 0.80 and in age > 40 years is 1.06	-Invasive carcinomas commonly affect the right-side breast at younger ages (< 40 years old).	-Higher frequency of histological types except invasive mucinous and medullary carcinomas -Right-handed patients find small lesions in left-side breast earlier.
Altundag K et al., 201 6[6].	-Left-handed patients had 2 years earlier onset of breast cancer -Right-handed patients had higher median age at diagnosis	Not Applicable	Not Applicable
Al Saad SK et al., 200 9[9].	Higher family history due to consanguineous marriage	Not Applicable	Not Applicable
Bao J et al., 201 4[11].	Unclear effect of laterality and primary tumor site	Not Applicable	Lower survival rates due to cardiac toxicity after radiotherapy
Fatima N et al., 201 3[12].	Higher laterality ratio (59%:41%; p < 0.001).	-More aggressive (Similar to our results) -Observed in younger age group (46:52 years; *p* < 0.0001) and with smaller primary tumor size -Higher negative receptor status and bone metastasis	Poor survival
Zeeneldin AA et al., 201 3[13].	Laterality ratio is 1.16	-More aggressive (similar to our results) -Higher positive HER2 and ductal cancers (similar to our results) -Higher in younger patients (similar to our results)	-Poor survival may be related to radiation toxicity -Higher lobular cancers
Dane S et al., 200 8[14].	Positive laterality	Frequent positive axillary lymph node metastasis (similar to our results)	Not Applicable
Amer MH, 201 4[15].	-Laterality ratio: 1.1 -Positive association between laterality and family history and age at diagnosis	Not Applicable	-Higher frequency of getting left-sided breast cancer in all ages except < 30 years, > 90 years and 50–59 years. -Left-sided disease was higher in Caucasian and African American ancestry.
King MC et al., 197 9[16].	No relation between family history and breast cancer laterality	Not Applicable	Not Applicable
Mokone-Fatunla DH et al., 201 9[17].	-Laterality ratio is 1.24 -Statistically significant association between laterality and stage (*p* = 0.050) -No statistically significant difference between age, site and histological type of breast cancer and laterality	Higher frequency of advanced cancer stages (similar to our results)	10.6% excess of left-sided breast cancer
Kakkar V et al., 202 0[18].	Reversed lateralization of breast cancer may be due to estrogen hormone	-More postmenopausal patients -Higher age at diagnosis > 50 years (not statistically significant) -Higher incidence in Indian populations	-More premenopausal patients -Not significant lower age at diagnosis < 50 years (similar to our results)
Sughue T et al., 201 4[19].	-Laterality ratio depends on birth country -Japan is 1.14 -Ryukyu Islands is 2.6 -Laos is 1.62 -Algeria is 2.1 -Poland is 0.92 -Overall SEER population is 1.04	Not Applicable	Statistically significant, increase in laterality ratio with age, and decrease with birth year and year of diagnosis (similar to our results)
Ing R et al., 197 7[20].	-Laterality ratio is 0.97 -Women who breastfed from one side developed cancer in the contralateral breast side with increased frequency in menopause	Higher in nulliparous women (similar to our results)	Higher frequency after menopause (55 years)
Safi T et al., 201 4[21].	12% excess of left-sided breast cancer	Not Applicable	Higher HER2, ER and PR positive receptors
Perkins CI et al., 200 4[22].	Positive laterality	Not Applicable	5% excess in left-sided breast cancer

Initial reports indicated a slightly worse prognosis in left-sided breast cancers, which was explained to be related to radiotherapy and its injurious effect to the myocardium in the left hemithorax [11**–**13]. This was not a universal finding in the subsequent reports, and the technical improvement of radiotherapy administration lowered radiotherapy-related cardiotoxicity in general [23].

In our study, the ratio of patients presenting with primary tumor size less than 5 cm was higher in the left-sided breast cancer group compared to the right-sided breast cancer group (88.7% versus 81.5%). Tumor size more than 5 cm was commoner in the right-side compared to the left-side (18.5% versus 11.3%). Other studies showed the same results as ours: a higher percentage of early-stage breast cancer in the left-side, and a higher percentage of advanced stage in right-side. The mechanism is not known. It has been postulated that right-handed patients examine their left-side breast and axilla more accurately than their right-side breast and axilla. This leads to an earlier discovery of smaller breast lesions in their left-side breast compared to the right-side breast [1**,** 2**,** 5]. It was also suggested that this might be the result of a stronger cell-mediated immune activity in the left-side of the human body [14].

In general, the ratio of positive family history of malignancy among our patients was high (33.6%), probably indicating a higher genetic influence. The popularity of consanguineous marriage might be a major contributing factor in this regard [9]. Positive family history was more prevalent in right-sided breast cancers (40.2%) than left-sided (27.8%). In contrast this, Amer et al. proposed a theory suggesting a relation between laterality and genetic predisposition, but when the clinical variables were studied, there was no relation between the variables and laterality [15]. Another study reported no relation between family history and laterality [16].

Studies on clinicopathological factors and their association to laterality are not high in number. In general, ipsilateral axillary lymph node metastasis, younger age at diagnosis, high tumor grade, positive family history, nulliparity, and advanced tumor stage are now well recognized clinicopathological factors associated with a lowered survival and a more aggressive breast cancer behavior [15**,** 16**,** 24].

In our study, positive ipsilateral axillary lymph nodes at presentation were higher in right-sided breast cancer, and this was consistent with the results of other studies [14**,** 25]. However, there was one study contradicting this fact as it showed that positive ipsilateral axillary lymph node involvement (N3, M0) in the left-sided breast cancer was associated with a shorter time to first metastasis, increased risk of distant metastases, and axial bone involvement when compared to right-sided breast cancers [26].

In our study, the low number of patients limits sound conclusions in relation to the effect of laterality on recurrence. Nevertheless, we observed a higher local recurrence ratio in the right-side (7%) compared to the left-side (4.5%). Distant metastases were more common in left-side (18%) compared to the right-side (10%).

Among our study group, the mean age of breast cancer at diagnosis was around 49 years. Right-sided breast cancers were more prevalent in the age group less than 50 years (55.6%). Similarly, a Chinese study showed a 10% higher probability of breast cancer diagnosis in the right-side breast than the left-side breast in 40 years of age and below, and also showed a 5% higher probability on the left-side breast compared to the right-side in the 40 years of age and above group of patients [5]. On the other hand, other studies showed no difference in laterality in relation to age at diagnosis [15**,** 17**,** 18]. In contrast to this, in a study from north India, Kakkar et al. found a higher ratio of left-sided breast cancer in the pre-menopausal group of patients, while the incidence of right-sided breast cancer was higher in the postmenopausal group of patients. A possible role of estrogen hormone level difference between each group was suggested as a possible explanation [18].

A study by Sughue et al. (USA) analyzed 1.2 million breast cancer cases between 1973 and 2010 and concluded that laterality ratio depends on the country of birth and not race. The study also found a statistically significant increase in laterality ratio with aging [19]. Our study also showed a similar trend of laterality increasing with age.

Although nulliparity was low in our study group (21.1%), we found nulliparity more prevalent in right-sided breast cancer (R 25%, L 17.4%) which was similar to a study from Hong Kong [20].

In our study, we found a statistically significant relation between tumor size of right-sided breast cancer and the five-year survival rate. This finding did not correlate with other studies stating that laterality is not related to prognosis in breast cancer [11].

An interesting study from Hong Kong on Chinese women showed that women who breastfeed from a single breast, like the right breast, developed cancer in the left breast only. This effect was perceived more during menopause (55 years & above) [20].

The association between laterality and type of breast cancer, if any, is still unclear. Our study has shown a higher percentage of IDC NST in right-sided breast cancers, while IDC ST like mucinous and medullary was higher in left-sided breast cancers. We also found predilection of DCIS in left-sided breast cancers. In view of the relatively low number of patients in our study, no sound observation can be made in regard to laterality and histological subtypes. It is worth mentioning here that a study from USA showed no significant laterality differences in invasive ductal or lobular carcinoma but there was left laterality in DCIS [2]. In contrast, another study showed left laterality in all subtypes of invasive breast cancer, with the exception of mucinous and medullary subtypes [5].

The molecular subtyping of breast cancer is currently a major factor in predicting the prognosis and tailoring the treatment [24]. A Lebanese study published in 2019 showed that HER2 overexpression, ER, and PR positivity were more frequent in left-sided breast cancers [21]. Our data is in short of making any suggestions in this regard.

Although our study showed that the overall 5-year survival rate was not statistically significant in relation to laterality per say, still right-sided breast cancers were significantly associated with the primary tumor size, and the stage of tumor. Similarly, few other studies reported more aggressive breast cancer features in tumors originating in the right breast [12**,** 13**,** 17].

### Limitations

Limitations of this study include the retrospective nature and the inclusion of patients operated by the authors only. The relatively small number of patients. This is understandable in view of the small population of Bahrain. However, the native Bahraini population is homogeneous, which reduces the resultant selection bias.

## Conclusion

This is the first study exploring the issue of breast cancer laterality in a defined Arabian population. Both Bahrain and Arabian Gulf countries share some demographic characteristics that differentiate them from western populations. The laterality ratio in this study was 1.06, which is consistent with the globally published range (1.05 to 1.26) and is increasing with increasing age.

In our study, statistical significance was reached. The right-sided breast cancers were associated with a higher positive family history of malignancy, a more locally advanced and metastatic disease at presentation, and a reduced 5-year survival in relation to size and stage.

Among our study group, younger age at diagnosis (less than 50 years), nulliparity, axillary lymph node metastases, IDC NST, HER2 overexpression, and local recurrence were more prevalent in right-sided breast cancers. These differences were not statistically significant. However, their potential indication toward a more aggressive tumor nature cannot be overlooked in view of the established evidence-based data in the literature.

The association between breast cancer laterality, and the hormonal and HER2 is still not widely addressed in the available literature, although other clinicopathological characteristics were extensively analyzed. Systemic reviews and meta-analyses are needed to answer the remaining unanswered question: is laterality in breast cancer still worth studying?

## Data Availability

The datasets generated and/or analysed during the current study are not publicly available due to patients’ privacy could be compromised but are available from the corresponding author on reasonable request.

## Abbreviations

**AGU**: Arabian Gulf University
**CMMS**: College of Medicine and Medical Sciences
**IDC**: Invasive Ductal Carcinoma
**NST**: No Special Type
**ST**: Special Type
**DCIS**: Ductal Carcinoma in situ
**ILC**: Invasive Lobular Carcinoma
**SD**: Standard Deviation
**ER**: Estrogen Receptors
**PR**: Progesterone Receptors
